# Vasospasm during Exertion: New Pathophysiological Insights

**DOI:** 10.5935/abc.20190090

**Published:** 2019-07

**Authors:** Gonzalo Navarrete, Eduardo Pozo, Fernando Rivero, Teresa Bastante, Luis Jesús Jiménez-Borreguero, Fernando Alfonso

**Affiliations:** Hospital Universitario La Princesa. Madrid, Madrid - Spain

**Keywords:** Coronary Vasoespasm/physiopathology, Coronary Vessels, Myocardial Ischemia, Coronary Angiography, Electrocardiology, Echocardiography

**Figure 1:**
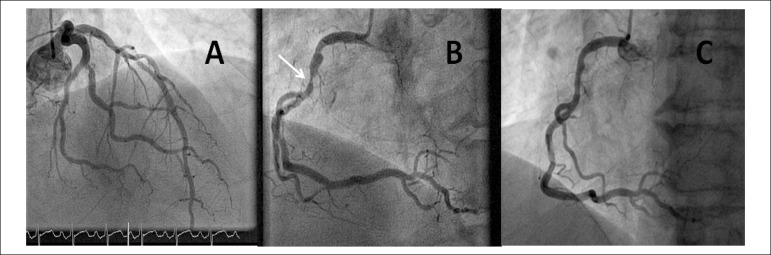
ICA after stress echocardiogram. A) ICA in caudal right oblique projection that demonstrates mild plaque in proximal LAD. B) ICA in left anterior oblique projection that showed severe obstruction in RCA (arrow). C) ICA in left anterior oblique projection after the implant of bioabsorbable scaffold in RCA. ICA: invasive coronary angiography LAD: left anterior descending artery. RCA: right coronary artery.

**Figure 2:**
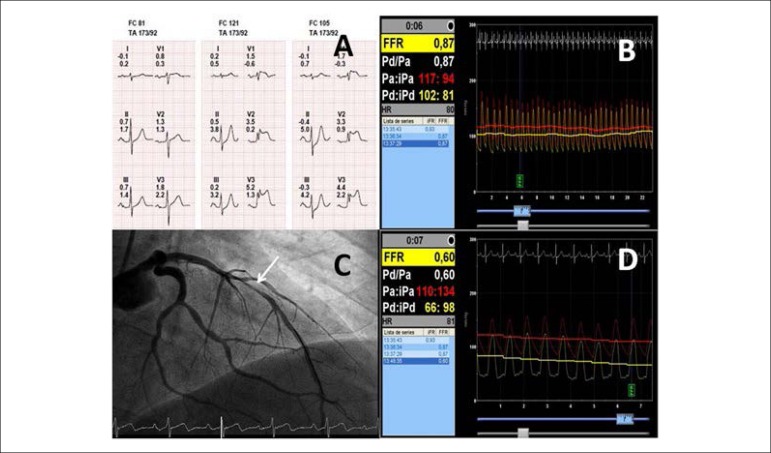
ECG during treadmill test, pressure guide pre and post vasospasm test, and ICA post vasospasm test. A) ECG during treadmill test showed ST elevation in V1-V4 leads. B) FFR value in proximal LAD which discarded functional significance of this lesion. C) ICA after methylergonovine injection confirmed focal vasospasm in LAD. Panel D: FFR value after methylergonovine injection that demonstrated functional significance of vasospasm. ICA: invasive coronary angiography; FFR: fractional flow reserve; LAD: left anterior descending artery.

A 69-year-old male with hypertension, dyslipemia and no previous cardiovascular disease presented a transient ST-segment elevation on electrographic (ECG) monitoring during a planned ureteroscopy. The patient remained asymptomatic during the postoperative period. He only referred very sporadic episodes of effort chest pain in the last years without progression or events at rest. Given the lack of ECG registry, an exercise stress echocardiogram was indicated. The study was clinical and echocardiographically positive for inducible ischemia, with hypokinesia of the mid and apical anterior wall segments at peak exercise without ECG changes. With the documentation of ischemia in the left anterior descending coronary artery (LAD) territory, a coronary angiogram was performed. Only a mild lesion in the proximal LAD was demonstrated. However, a severe lesion in the proximal right coronary artery (RCA) (Figure1A and 1B), was treated with a bioabsorbable scaffold (Figure 1C), despite the absence of inducible ischemia demonstration in that territory. The patient was discharged on dual antiplatelet treatment, betablockers and statins and remained asymptomatic without new episodes of chest pain. Six months later, during another ureteroscopy, a new episode of transient ST-segment elevation was observed on ECG monitoring. At that time, a treadmill test was performed showing marked transient ST-segment elevation in V1-V4 leads (Figure 2A). Repeated coronary angiography demonstrated the good result of the previously implanted stent in RCA and persistence of a mild plaque in the proximal LAD. Due to the previously documented ECG changes in the right precordial leads, a pressure guide was performed in the proximal LAD, which ruled out the functional significance of this lesion (iFR 0.88; FFR 0.87 with 600 mcg Adenosine) (Figure 2B). In the light of the discordant ECG, echocardiographic and angiographic findings, a vasospasm test with intracoronary methylergonovin (20 mcg) was eventually carried out. This test was positive with angina reproduction and a significant reduction in LAD vessel diameter up to 90% diameter stenosis (Figure 2C) with a concomitant Pd/Pa value of 0.60 (Figure 2D). Finally, the patient could be discharged under treatment with nitrates and calcium channel blockers, remaining asymptomatic at 1-year follow-up. An exercise stress echocardiogram was negative for inducible ischemia and repeated ureteroscopy procedures were uneventful.

Coronary artery spasm (CAS) that clinically represents variant or Printzmetal angina,^1^ is caused by a sudden an intense vasoconstriction of an epicardial coronary artery, which can appear both at the site of stenosis and in angiographically normal coronary arteries. CAS during exertion is an atypical presentation of this clinical entity, firstly described by Cheng et al in 1973, as the “variant of the variant”.^2^ The pathophysiology of CAS during exercise has not been completely clarified yet. However, it is well established that exercise can induce CAS in patients with variant angina, as demonstrated in small groups of patients subjected to supine bicycle exercise on the cardiac catheterization table.^3^ Exercise stress echocardiogram induced ST-segment elevation with striking discordance between the echocardiographic and angiographic findings is a rare condition that may be a result of severe CAS. To our knowledge, however, echocardiographic abnormalities unraveled during exercise echocardiography in patients with variant angina have not been previously described. Furthermore, there is no previous evidence of the potential value of physiological assessment of lesion severity, with pressure wire quantification of FFR and iFR, during CAS induction. Physiological assessment of the dynamic changes detected on Pd/Pa immediately after methylergonovine injection may be of potential clinical value in this vexing clinical setting.

This case represents an atypical presentation of variant angina in a patient with coronary artery disease. We hypothesize that hyperventilation, both during exercise and during anesthesia, might act as a trigger for CAS. In these situations, ischemia was nicely shown with noninvasive (exercise stress echocardiogram) as well as invasive diagnostic techniques (methylergonovine test). Provocative spam testing continues to be a clinically useful tool to diagnose vasospastic angina validated in previous studies with a sensitivity of ≥ 90% and specificity ≥ 97%.^4^ In summary, our findings illustrate the possibility of CAS even in patients with significant coronary artery disease and exertional chest pain. Further diagnostic insights are of major clinical value when discordance between electrocardiographic, echocardiographic and angiographic findings persists.
